# Retrospective validation of the postnatal growth and retinopathy of prematurity criteria in a Chinese cohort

**DOI:** 10.3389/fped.2025.1509106

**Published:** 2025-06-04

**Authors:** Li Li, Yanlin Gao, Yuhan Lu, Wei Chen, Mei Han

**Affiliations:** ^1^ Tianjin Eye Hospital, Tianjin Key Laboratory of Ophthalmology and Visual Science, Tianjin, China; ^2^ School of Medicine, Nankai University, Tianjin, China

**Keywords:** retinopathy of prematurity, postnatal growth and retinopathy of prematurity, G-ROP, screening, prediction model

## Abstract

**Purpose:**

To evaluate the effectiveness and applicability of the postnatal growth and retinopathy of prematurity (G-ROP) screening criteria in a Chinese neonatal cohort.

**Methods:**

Data pertaining to the retinal screening of premature infants admitted to the neonatal intensive care unit from January 2021 through December 2021 were retrospectively analyzed. The severity of ROP was graded on the basis of the International Classification of Retinopathy of Prematurity criteria established in 2005. Treatment decisions for ROP were guided by the recommendations of the Early Treatment for Retinopathy of Prematurity Cooperative Group. The presence of six key variables that comprise the G-ROP screening criteria were carefully documented. The sensitivity and specificity of the G-ROP predictive algorithm in identifying infants with ROP requiring treatment were calculated.

**Results:**

A total of 352 infants with complete records were included in this study, among whom 120 infants (34.1%) were diagnosed with ROP. Of those, 21 infants (6.0%) received treatment. By applying the 6 criteria of the G-ROP model, all infants with severe ROP were successfully identified. The sensitivity of the G-ROP model in predicting treatment-requiring ROP was 100% (CI, 0.808–1.00), and the specificity was 47.8% (CI, 0.413–0.545). By applying the G-ROP criteria, the number of infants who required ROP screening would have been reduced by 122 (34.7%), while the number of screenings (1967) would have been reduced by 537 (27.3%).

**Conclusion:**

The prevalence of ROP (34.1%) and treatment-requiring ROP (6.0%) were relatively high in our cohort. Application of the G-ROP prediction model can improve the sensitivity and specificity of ROP screening. All infants with treatment-requiring ROP were correctly identified. The G-ROP screening criteria seemed to be effective and appropriate for predicting ROP in infants living in Tianjin, China.

## Introduction

Retinopathy of prematurity (ROP) is a pathophysiological disorder characterized by the dysregulated proliferation of retinal blood vessels in premature infants with incomplete retinal vascularization. The aberrant angiogenesis that characterizes ROP can lead to severe vision impairment or blindness if not appropriately managed; therefore early screening and timely intervention for at-risk populations is highly important (1, 2).

Early intervention, which can be achieved through laser photocoagulation therapy or intravitreal administration of anti-vascular endothelial growth factor (anti-VEGF) agents, serves as a critical preventative measure against the progression of ROP (3). Therefore, it is important to initiate timely screening protocols for this ocular pathology. Currently, the criteria adopted internationally for identifying individuals with ROP are predominantly based on gestational age (GA) and birth weight (BW) as pivotal risk indicators (4–6). Although these parameters demonstrate desirable sensitivity in forecasting severe ROP, their specificity is suboptimal. Notably, the percentage of infants requiring therapeutic intervention in accordance with these criteria remains below 10% across countries worldwide, including China (7–9), the United States (10), and the United Kingdom (11). Contemporary studies have advocated the use of the postnatal growth and ROP (G-ROP) screening criteria as the gold standard model (12, 13). Improving this screening algorithm could substantially mitigate the considerable logistical and financial burdens associated with the extensive screening of premature neonates for ROP.

In the present study, our objective was to extensively evaluate the sensitivity and specificity of the G-ROP screening criteria in order to verify the clinical utility of the G-ROP protocol for the early identification of ROP within the healthcare system of Tianjin, China. We further sought to introduce a novel paradigm in ROP surveillance that could replace the conventional reliance on GA and BW alone, thereby improving the precision and efficacy of ROP screening strategies in regional healthcare systems.

## Patients and data collection

This study involved the retrospective analysis of data collected at the neonatal intensive care unit of Tianjin Eye Hospital from January to December 2021. This study adhered to the tenets of the Declaration of Helsinki and was approved by the Ethics Committee of Tianjin Eye Hospital. Written informed consent was obtained from all the infants' parents or guardians. All methods were carried out in accordance with the relevant guidelines and regulations. The BWs and GAs of the infants were recorded at each fundus examination. Daily BW was reported by the infants' parents or guardians, and postnatal weight gains were subsequently calculated. Over the course of one year, all preterm infants who underwent ROP screening were included in the study. Infants were excluded if they had incomplete data, no ROP screening results, or any retinal vascular diseases other than ROP.

The G-ROP model is based on the assessment of 6 criteria (14–16): GA < 28 weeks, BW < 1,051 g, weight gain < 120 g from 10–19 days PNA, weight gain < 180 g from 20–29 days PNA, weight gain < 170 g from 30–39 days PNA, or hydrocephalus.

## Screening criteria and diagnosis

The fundus examinations were carried out according to the ROP guidelines recommended by the Chinese Ophthalmological Society in 2014: (6) infants with a GA < 32 weeks and/or BW < 2,000 g or who were suspected to be at risk of ROP (such as those who received long-term oxygen supplementation or with serious systemic diseases).

Fundus examinations were performed by an experienced ophthalmologist with a RetCam III digital camera (Clarity Medical Systems, USA). The stages of ROP were determined on the basis of the International Classification of Retinopathy of Prematurity (2005) (17). If different stages were recorded for the same eye, the most severe stage of ROP was recorded. ROP was divided into two types: Type 1, which includes stage 1 or 2 ROP in Zone I with plus disease, stage 3 ROP in Zone I with or without plus disease, or stage 2 or 3 ROP in Zone II with plus disease; and Type 2, which includes stage 1 or 2 ROP in Zone I without plus disease or stage 3 ROP in Zone II regardless of the presence of plus disease. Moreover, according to the extent of the lesions, ROP was divided into mild ROP, which includes Type 2 and milder ROP, and severe ROP, which includes Type 1 and more severe ROP as well as aggressive posterior ROP (AP-ROP) and is an indication for mandatory treatment, in accordance with the Early Treatment for Retinopathy of Prematurity (ETROP) Study (18). Treatment was carried out within 72 h when severe ROP was detected.

## Statistical analysis

The performance of the G-ROP criteria was determined by calculating the sensitivity and specificity in predicting treatment-requiring ROP. The GA and BW of the infants were compared among groups with the Kruskal–Wallis H test. Sex distributions and the prevalence of multiple births were compared between groups using chi-square tests. The statistical analyses were performed with SPSS (version 19.0; SPSS Inc., Chicago, IL), and **95%** confidence intervals (CIs) were calculated using the Wilson score method. *P* < 0.05 was considered to indicate statistical significance.

## Results

### Subject characteristics

A total of 365 preterm infants were hospitalized from January 2021 to December 2021. Of these, 13 infants were excluded due to loss to follow-up. Therefore, 352 infants with available screening data were included in the G-ROP model analysis. The mean GA was 30.2 ± 2.7 weeks, and the GA ranged from 25 weeks–35 weeks. The mean BW was 1,220.0 ± 353. 3 g, and the range of BW was 650 g to 2,100 g. 195 infants (55.4%) were males, and 82 infants (23.3%) were the results of a multiple birth.

The screening data for retinopathy of prematurity are shown in Table 1. A total of 120 infants (34.1%) were diagnosed with ROP. GA and BW were significantly different between infants with and without ROP (27.9 ± 2.4 w vs. 31.4 ± 2.1 w, *p* < 0.001; 1,050.7 ± 270.1 g vs. 1,307.6 ± 359.9 g, *p* < 0.001, respectively). Sex and the number of infants who were the result of a multiple birth were not different between infants with and without ROP.

**Table 1:** Characteristics of the study infants.

Characteristic	No ROP	ROP	Mild ROP	Severe ROP	*P* value (No ROP vs. ROP)
Number (%)	232 (65.9)	120 (34.1)	99 (28.1)	21 (6.0)	NA
GA, mean ± SD, weeks	31.4 ± 2.1	27.9 ± 2.4	28.3 ± 2.0	26.6 ± 1.6	<0.001
(range, weeks)	(27–35)	(25–35)	(26–34)	(25–28)	NA
BW, mean ± SD, g	1,307.6 ± 359.9	1,050.7 ± 270.1	1,128.5 ± 241.2	920.5 ± 109.2	<0.001
(range, weeks)	(650–2,100)	(670–2,100)	(740–2,020)	(670–1,100)	NA
Gender (male: female)	130:102	65:55	50:49	15:6	0.738
Multiple birth	51	31	28	3	0.418

Among 352 premature infants, 99 (28.1%) were diagnosed with mild ROP, and 21 (6%) were diagnosed with severe ROP. No children experienced AP-ROP during our study. All 21 infants with severe ROP were treated promptly within 72 h.

Among the six criteria of the G-ROP model applied in this study, hydrocephalus was not identified in any of the infants. A total of 230 infants met at least one of the other five criteria (Table 2). Among these, 121 infants had not been diagnosed with ROP, while 88 and 21 infants had been diagnosed with mild ROP and severe ROP, respectively. All infants with severe ROP were identified with at least one of the six criteria of the G-ROP model. Among these, 19 infants with severe ROP were identified by a BW < 1,051 g and/or a GA < 28 weeks, and the other 2 infants were identified by weight gain < 180 g from 20–29 days PNA and weight gain < 170 g from 30–39 days PNA.

**Table 2:** Prediction of ROP with the G-ROP screening criteria and the infant characteristics.

Sensitivity of model	No ROP	ROP	Mild ROP	Severe ROP
Number	232	120	99	21
Trigger+ (%)	121 (52.2)	109 (90.8)	88 (88.9)	21 (100)
Trigger− (%)	111 (47.8)	11 (9.2)	11 (11.1)	0 (0)
Sensitivity (95% CI)	NA	90.8%	88.9%	100.0%
Specificity (95% CI)	47.8%	NA	NA	NA
Reduction in the number of infants who had been identified with ROP screening (%)	111 (47.8)	11 (9.2)	11 (11.1)	0 (0)

The sensitivity of the G-ROP model in predicting ROP was 90.8% (95% CI, 0.828–0.951), and the specificity was 47.8% (95% CI, 0.413–0.545). The sensitivity of the G-ROP model in predicting severe ROP was 100% (95% CI, 0.808-1). Applying the G-ROP algorithm would have prevented ROP screening for 122 infants. Of these, 11 infants eventually developed mild ROP, but none of them required any treatment. These results suggested that the G-ROP algorithm is safe and effective in reducing the need for ROP screening by 34.7%. Moreover, use of the G-ROP algorithm would have reduced the total number of ROP screenings (1967) by 537 (27.3%).

## Discussion

At present, countries worldwide use GA and BW, the most important and highly sensitive risk factors for ROP, as mandatory criteria when screening for the disease. Indeed, the corresponding criteria cover almost all severe cases of ROP. However, due to insufficient specificity, the extent of screening is often expanded so that ROP children who need treatment are not missed; as a result, however, the frequency of unnecessary screening is elevated. Some guidelines indicate that “newborns with unstable overall physical conditions after birth”, including infants of a certain GA and those with relatively high birth weights that may predispose them to developing ROP, should also be screened. Therefore, some other postpartum factors must affect patient outcomes, and new indicators need to be added to properly limit the scope of screening.

In recent years, an increasing number of researchers have shown interest in the correlation between postnatal weight gain and the occurrence of ROP, establishing ROP prediction models based on basic indicators including GA, BW, and postnatal weight gain (19, 20). These models include the WINROP (21), CHOP ROP (22), CO-ROP (23), and G-ROP (12, 16). Among them, the G-ROP is currently the only multicenter, large sample prediction model that is widely used in many ROP studies.

The G-ROP model has high sensitivity in predicting the ROP in individuals from developed countries. However, there are few studies on the effectiveness and sensitivity of the G-ROP model in the Chinese ROP population. We retrospectively analyzed the ROP screening data from the largest NICU in Tianjin to validate the performance of the G-ROP screening criteria.

In this study, the sensitivity of the G-ROP model in predicting ROP was 90.8%, and the specificity was 47.8%. Furthermore, the sensitivity in predicting mild ROP was 88.9%, and that in predicting severe ROP was 100%. All infants with severe ROP were correctly identified by the G-ROP algorithm.

Nineteen of the 21 infants with severe ROP met the GA or BW criteria of the G-ROP model, indicating that low GA and BW are still the most important risk factors for the development of ROP. The GA and BW of the other 2 infants were 29 weeks and 1,080 g and 28 weeks, 1,100 g, respectively. Only 2 infants (0.6%) with relatively high GA and BW were identified by the weight gain criterion of the G-ROP model. Although the sensitivity of the model was very low, the weight gain criterion of the G-ROP model remains necessary, as it can identify due to its role in severe ROP. Applying the G-ROP screening criteria would reduce the number of screened infants by 34.7%. Among these infants, only 9% with mild ROP would have been excluded by the G-ROP model. These results are similar to those of previous studies investigating the performance of the G-ROP algorithm (14–16, 24). All indicators are tracked until 39 days PNA on the basis of the G-ROP screening criteria. Consequently, the number of ROP screenings would be reduced by 10.5% because of the shorter ROP screening period. Therefore, applying the G-ROP screening model could help in the timely diagnosis of severe ROP and reduce unnecessary physiological pressure and the family medical burden for low-risk infants.

As stated previously, GA and BW are major risk factors for the development of ROP. Other factors, such as the level of neonatal care level, the extent of oxygen supplementation, serious systemic diseases, and long-term hospitalization, have also been considered to be associated with ROP development (25). Because of these risk factors, some infants with higher GA and BW underwent ROP screening. The ROP screening criteria implemented in different countries are mainly based on BW and GA. These ROP screening criteria typically show high sensitivity but low specificity, partially due to the low incidence of severe ROP. In European and American countries, the incidence of severe ROP ranges from 6.1%–14.3% (26–29), whereas in China, the incidence ranges from 1.1%–28.3% (7–9, 30, 31). Compared with those in developed regions, a greater number of older premature infants tend to experience more severe ROP in remote areas and economically underdeveloped regions (32). The World Health Organization reported that the mean BW of infants with severe ROP was 750 g in developed countries but 1,500 g in developing countries (33). Fundus examinations for infants with a GA > 32 w are not recommended according to the ROP screening guidelines of developed countries (34, 35), whereas severe ROP in infants with a GA > 32 weeks is not uncommon in developing countries (8, 36). However, although implementing wide-scale ROP screening would avoid missing infants with treatment-requiring ROP, it would also lead to an increase in the number of screened infants and longer examination times. The incidence of severe ROP in this study, 6.0%, was higher than that reported in a previous study in Tianjin, at 1.9% (7). Infants hospitalized in the NICU are more likely to have been born preterm and to have a lower BW or severe systemic diseases. These disadvantageous factors could have led to the higher incidence of severe ROP in this study.

The optimal screening criteria should minimize the number of infants screened as well as the number of ROP screenings. Furthermore, no infants needing treatment should be missed. The G-ROP model has high sensitivity in developed countries. Binenbaum et al. reported that in a multicenter, retrospective cohort study of infants undergoing ROP screening at 29 hospitals in the U.S. and Canada from 2006–2012, the G-ROP model successfully predicted Type 1 ROP in 100% of infants and would reduce the number of infants who should undergo screening by 30%. Moreover, the sensitivity of the G-ROP model was greater than that of the U.S. ROP screening guidelines (99.3%) (12). Vinayahalingam et al. retrospectively validated the postnatal growth and G-ROP criteria in Switzerland from 2015–2019. The sensitivity in predicting treatment-requiring ROP was 100%, and the specificity was 41%. Applying the G-ROP screening criteria could have reduced the number of infants requiring ROP screening by approximately one-third (24).

Nevertheless, the sensitivity of the G-ROP prediction model is low in developing countries. In a retrospective Turkish study, the sensitivity and specificity of the G-ROP model were 91.2% and 34.1%, respectively, in identifying treated ROP patients. The incorporation of bronchopulmonary dysplasia into the G-ROP model increased the sensitivity to 100% and reduced the number of infants who would require screening by 22.7% (15). Despite China's status as a developing nation, the application of the G-ROP model in our study yielded sensitivity and specificity metrics comparable to those observed in North America and surpassing the findings from Turkey. This outcome was likely attributable to Tianjin's comparatively greater economic status, its advanced healthcare infrastructure, and its **ROP detection levels similar to those of developed countries.** In retrospective G-ROP studies conducted in the U.S. and Canada, only 233 (3.1%) out of 7,438 infants (37) and 120 (3.0%) out of 3,981 infants (16), respectively, included as the study cohorts were of Asian descent. Therefore, the generalizability of the G-ROP screening criteria in Asian infants should be evaluated in larger Asian study populations. In Japan, Shiraki et al. reviewed 537 premature infants with complete ROP screening outcomes and weight gain data. Of these, 81 infants required treatment for ROP, and 218 infants did not, and the G-ROP model reached a sensitivity of 100% and a specificity of 28.9%. Use of the model would have reduced the percentages of infants requiring ROP screening and fundus examinations by 24.5% and 12.9%, respectively (14). Therefore, the G-ROP screening criteria seemed to be more effective for infants in Tianjin than for those in Japan. Huang et al. validated the G-ROP screening criteria in a Taiwanese cohort (38). Among the 303 included infants, 103 developed ROP, 29 of whom developed Type 1 ROP. The sensitivity and specificity of the G-ROP model were 96.6% and 42.3%, respectively, in the detection of type 1 ROP. Applying the G-ROP criteria would have reduced the number of infants requiring ROP screening and fundus examinations by 32.6% and 33.5%, respectively. These findings are similar to those of our study in Tianjin infants. In a Saudi Arabian cohort of 300 preterm infants, 30 infants developed severe ROP (10%), and 85 infants had mild ROP (28.3%). The sensitivities of the G-ROP 1 and G-ROP 2 models (with identical criteria as the G-ROP 1 model except for weight gain, whose thresholds were set to <180 g) for severe ROP were 96.7% and 100%, respectively, and the specificities were 24.4% and 16.7%, respectively. Use of the G-ROP 2 model would have reduced the number of screened infants by 15% (39). Yang Lu et al. included 1,634 premature infants from two research centers in Zhejiang Province; according to the Chinese ROP screening criteria, 25 infants had severe ROP, and 399 had mild ROP. Moreover, 844 premature infants met the G-ROP criteria; consequently, the G-ROP model had a specificity of 35.0% and a high (96.0%) but not perfect sensitivity in predicting severe ROP (40).

The low sensitivity of the G-ROP model in developing countries may be due to the following reasons:

1. Limitations in medical resources and access to suitable technologies: Developing countries are relatively lacking in medical resources and have outdated equipment for premature infant care and monitoring, and the medical personnel tend to lack suitable levels of experience, all of which affect the collection of premature infant-related data, assessment of the infants' conditions, and timely and accurate diagnosis, thereby affecting the sensitivity of the model.
2. Differences in the baseline conditions of premature infants: Due to economic conditions, nutritional status, healthcare awareness, supplemental oxygen use, and environmental factors such as hygiene and air pollution, the baseline conditions of premature infants, such as BW and GA, in developing countries may differ from those in developed countries. For example, developing countries may be more likely to have low birth weight infants, extremely premature infants, or gross full-term infants who also develop ROP-like lesions. These children's conditions may be more complex than those of infants from developed countries, and the infants are more prone to various complications and comorbidities, such as infections and respiratory distress syndrome. These diseases increase the risk and complexity of retinopathy in premature infants and may not be fully accounted for in the G-ROP model, making it difficult for the model to accurately reflect the conditions of the patients and reducing prediction sensitivity.

As mentioned above, many risk factors affect the occurrence of ROP, including maternal factors, factors that emerge in the prenatal and perinatal periods, neonatal monitoring levels, supplemental oxygen use, neonatal complications, and genetic, social, medical, and environmental conditions. These factors are not thoroughly included in the G-ROP model, and so a more comprehensive and authoritative screening model needs to be established. However, the relative simplicity of the G-ROP model makes it highly convenient for implementation in clinical practice.

There are several limitations to this study. First, this was a retrospective study with a small, single-center sample. The results of this study need to be verified in further multicenter and larger cohort studies. Second, no premature infants with hydrocephalus were included in this study, likely because of the low prevalence of the condition. The performance of the G-ROP model in predicting ROP in these patients needs to be validated. Previous studies have shown that oxygen supplementation has a greater effect on the development of ROP than does postnatal weight gain (13, 41, 42). The effect of excessive oxygen supplementation on the performance of the G-ROP model, however, has not been evaluated. In addition, as Tianjin is considered one of the more developed cities in China, the performance of the G-ROP screening criteria still needs to be verified in multiple, undeveloped regions of the country.

## Conclusion

In conclusion, the prevalence of ROP (34.1%) and treatment-requiring ROP (6.0%) were high in premature infants hospitalized in the NICU. Applying the G-ROP prediction model could improve the sensitivity and specificity of ROP screening. Most importantly, all treatment-requiring ROP infants were correctly identified. The number of infants requiring ROP screening would be reduced by 34.7% with use of the model. These findings suggest that the G-ROP model has strong performance and seems effective and appropriate for identifying ROP in infants from Tianjin.

## Data Availability

The original contributions presented in the study are included in the article/Supplementary Material, further inquiries can be directed to the corresponding author.
